# Experimental study on the electrohydrodynamic deformation of droplets in a combined DC electric field and shear flow field

**DOI:** 10.1016/j.fmre.2021.10.011

**Published:** 2021-12-22

**Authors:** Xiangdong Liu, Guanqiu Hao, Bo Li, Yongping Chen

**Affiliations:** a Key Laboratory of Energy Thermal Conversion and Control of Ministry of Education, School of Energy and Environment, Southeast University, Nanjing 210096, China; b School of Electrical, Energy and Power Engineering, Yangzhou University, Yangzhou 225009, China; c Laser Fusion Research Center, China Academy of Engineering Physics, Mianyang 621900, China

**Keywords:** Droplet deformation, Electrohydrodynamics, Electric field, Shear flow, Prediction model

## Abstract

A visualization experiment was conducted to investigate the electrohydrodynamic deformation of droplets in a combined DC electric field and shear flow field. Detailed experimental data on both the transient and steady droplet deformation parameters (*D*) and orientations (*ϕ_d_*) are provided at *R* > *S* and *R* < *S* (*R*: conductivity ratio; *S*: permittivity ratio) under different electric field and shear flow field combinations. The internal flow characteristics of the deformed droplet were also examined via the digital particle image velocimetry (DPIV) method. Due to the competition of the extensional component (EC) and the rotational component (RC) of these two fields on the droplet, the response of *ϕ_d_* is faster than that of *D* when an electric field is combined with a shear flow. Additionally, under the competition of the EC and RC at *R* > *S* and *R* < *S*, the steady-state *D* and *ϕ_d_* values exhibit distinct variations. In particular, surface charge convection plays a non-negligible role in enhancing and reducing droplet deformation at *R* > *S* and *R* < *S*, respectively. In addition, an asymmetric vortex forms inside the deformed droplet in the combined fields, and its velocity is lower under *R* > *S* and higher under *R* < *S* than in pure shear flow. The available prediction models use the experimental data to predict *D*, and a modified prediction model is proposed for improving the prediction accuracy of *ϕ_d_*.

## Highlights

• Electrohydrodynamic deformation of droplet in combined electric field and shear flow is studied.
• Internal flow of the deformed droplet is quantitatively examined via the DPIV method.
• Competition of actions from electric field and shear flow on droplet deformation is clarified.
• Prediction model is modified and demonstrated to quantitatively represent droplet deformation.

## Graphical abstract

**Figure:**
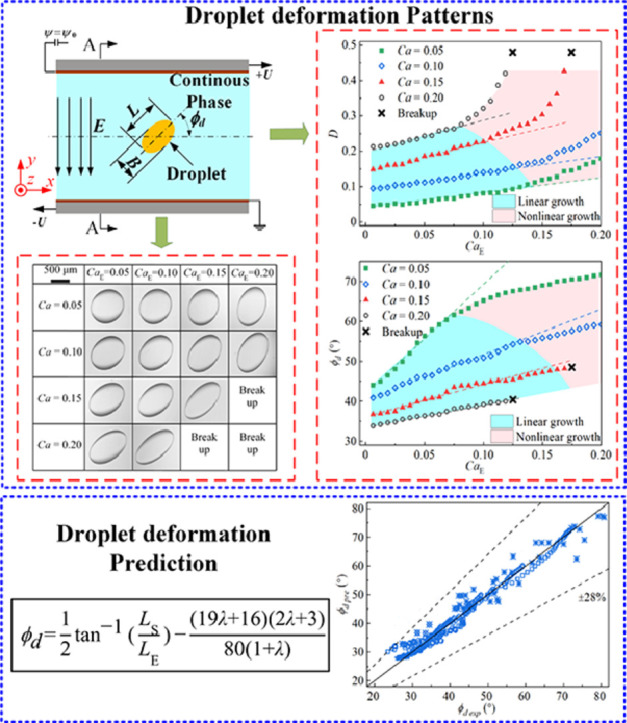

## 1 Introduction

Due to an insulation structure enclosed by an interface, as well as a tiny volume and high specific surface area, droplets are regarded as promising candidates as unique "reactors" for the mixture [1], analysis [2] and transport of reagents [3]. Thus, droplets have promising application prospects in the fields of energy, biology, chemistry, and pharmaceuticals, including as inertial confinement fusion target microspheres [4] and in the extracellular regulation of signal transmission [5], emulsification [6], and targeted drug delivery [7]. It is widely acknowledged that the performance of droplets in these applications is guaranteed by the precise control of droplet hydrodynamic behaviors (e.g., trajectory, deformation, breaking up, and coalescence) via external physical fields. In this context, understanding the mechanisms underlying droplet hydrodynamic behaviors when subjected to external physical fields is of great significance.

Droplets will inevitably be subjected to external flow fields in the above mentioned applications. To scientifically and quantitatively study the droplet hydrodynamic characteristics, controllable linear flow fields are usually used as the external flow field, with the linear shear flow field being the most common [8]. Studies on droplet hydrodynamics in shear flow fields were pioneered by Taylor [9]. It was shown that due to the competition between viscous stress from shear flow and interface tension, droplet shapes were changed from spherical to ellipsoidal, with the major axis inclined at an angle relative to the direction of the external unconfined shear flows. In particular, Taylor [9] proposed a formula for predicting the extent of small deformations as a function of the viscosity ratio and capillary number. Then, Torza et al. [10] and Guido and Simeone [11] provided experimental results that validated the theoretical formula for predicting droplet deformation in shear flow fields. In addition, it was found that [12] when the droplet is subjected to a confined shear flow, wall effects cannot be neglected, which promotes steady droplet deformation and induces complex deformation oscillations [13]. To predict the degree of steady droplet deformation in a confined shear flow, Shapira and Haber [14] derived a theoretical formula based on Lorentz's reflection method [15]. Further experimental studies were conducted to acquire more droplet deformation data under confined shear flows, which was then used to modify the prediction formula [13,[16], [17], [18]]. In summary, to date, droplet deformation hydrodynamics in pure shear flow have been extensively investigated. In particular, prediction formulas for the degree of steady deformation have been thoroughly developed and are regarded as reliable and effective theoretical foundations for droplet deformation fluidic control via external flow fields.

At present, there is an increasing demand for precise control droplet applications, necessitating a higher level of control accuracy and efficiency to achieve the designed interface droplet structure. However, the abovementioned fluidic control of droplet deformation via pure external flow fields is usually passive, unresponsive and difficult to control, and it needs to be improved to meet the growing demands for highly accurate and efficient control. In addition, for a given continuous phase and droplet phase with fixed properties, the deformation parameter and tilt angle of a deformed droplet in pure external shear flow are closely linked, and both are determined by the external shear strength. For example, a droplet with an unchanged deformation parameter cannot be rotated by a pure shear flow field. It should be noted that some other external physical fields, including electric fields, magnetic fields, and ultrasonic fields, could be used as candidates for assisting in the common pure fluidic control of droplets. In particular, electric fields are regarded as effective options for controlling droplet hydrodynamic behaviors because they are widely applicable, respond rapidly, and can be precisely adjusted [19,20]. Therefore, an electric field can be used to impose electric stresses in the normal direction of the droplet interface, which can increase the degree of manipulation freedom and thus amplify the control options and efficiency.

The electrohydrodynamic deformation of droplets suspended in an immiscible continuous fluid subjected to a combined external flow field and an electric field has aroused renewed interest in recent years. A few decades ago, Allan and Mason [21] first carried out an experiment on the deformation characteristics of droplets under combined shear and electric fields that tested the deformation degree and tilt angles of perfectly conductive droplets and weakly conductive droplets suspended in a weakly conductive continuous phase. In particular, based on a superposition of the electric and shear deformation forces, they proposed a theory for predicting the droplet deformation degree and orientation. However, due to the limitations of the experimental conditions, very few data were reported by Allan and Mason [21] without providing detailed experimental parameters. Moreover, their prediction theory assumed that the droplet was either a perfect conductor or an ideal dielectric, which led to appreciable prediction deviations from real droplet deformations that could not explain the oblate deformation of the droplet. Years later, Ha and Yang [22] investigated the deformation and orientation characteristics of a liquid capsule subjected to a coupled linear shear flow and a uniform direct current (DC) electric field and analyzed the effects of physical parameters (i.e., surface viscosity and viscosity ratio) on the orientation of the liquid capsule. In particular, based on the classical leaky dielectric model by Taylor [23], which states that perfect conductors and ideal dielectrics have finite permittivities and conductivities, various droplet deformation shapes could be reasonably explained in their work. However, quantitative deformation degree data were still lacking in their work. In addition, Feng [24] found that in leaky dielectrics, tangential traction is generated by the interaction between the electric field and the accumulated charges at the interface, which induces neighboring fluids to move and causes so-called surface charge convection (SCC). SCC has the ability to enhance prolate deformation, especially at large deformation degrees, which is not considered in the classical leaky dielectric model but cannot be neglected in the prediction of droplet deformations in electric fields [25]. To date, there are still limited effective experimental droplet deformation data under a combined electric field and external flow field.

In addition to the limited experimental research described above, an increasing number of numerical and analytical studies have been performed to study the electrohydrodynamic deformation of droplets under a combined external flow and electric field [26]. For example, Mählmann and Papageorgiou [27] simulated and compared the deformation difference of a droplet as a perfect dielectric and an ideal leaky dielectric, which showed that the electric field increased droplet elongation and rotated the droplet to the direction of the electric field for both the perfect dielectric and the leaky dielectric. This force brings the droplet closer to the shear wall, which exerts a stronger shear force, promoting droplet deformation. Vlahovska [25] first considered SCC under a large viscosity ratio of the droplet to the continuous phase and proposed a mathematical analysis based on the Ohmic and Stokes equations to describe fluid motion during droplet deformation. It was found that SCC due to the external shear flow affected both the normal stress and the shear viscosity. Afterward, using different interface tracking/capturing methods (e.g., VOF, phase-field, lattice Boltzmann method), Chakraborty's [28,29] and Biswas's groups [30] performed various numerical simulations on droplet deformation in shear [29,31], extensional [32], and Poiseuille flow [33,34] in the presence of a DC electric field. In particular, Mandal and Chakraborty [29] clearly demonstrated that an electric field perpendicular to the direction of shear flow changed not only droplet morphology but also droplet rheological properties. In addition to considering SCC under a large viscosity ratios, they also considered SCC under weak flows (*Ca* ≪ 1) and proposed a modified analytical solution for predicting droplet deformation behaviors. Unfortunately, when the SCC is strong and the droplet deformation is large, the predicted results from their model deviated considerably from the experimental data.

In summary, considerable attention has been paid to the electrohydrodynamics of droplet deformation in combined electric fields and external flows. However, there is still a lack of effective experimental data. Although increasing numerical and theoretical efforts have been devoted to this topic in recent years, the available prediction models for deformation behaviors need to be improved, especially when the SCC is strong and the droplet deformation is large, as in shear flow. Therefore, to provide effective experimental data, reveal the mechanism underlying droplet electrohydrodynamic deformation, and pursue approaches for predicting deformation characteristics, herein, a visualization experiment was conducted to investigate the electrohydrodynamic deformation of droplets in a combined DC electric field and shear flow. Detailed experimental data on both the transient and steady-state droplet deformation degree and orientation are provided. The internal flow characteristics of the deformed droplet were examined with the DPIV method. In addition, considering considerable SCC and a high deformation degree, the available prediction models use the experimental data are used to predict the droplet deformation degree, and a modified prediction model is proposed for improving the tilt angle prediction accuracy.

## 2 Description of experiment system

### 2.1 Experimental setup

**Fig. 1:**
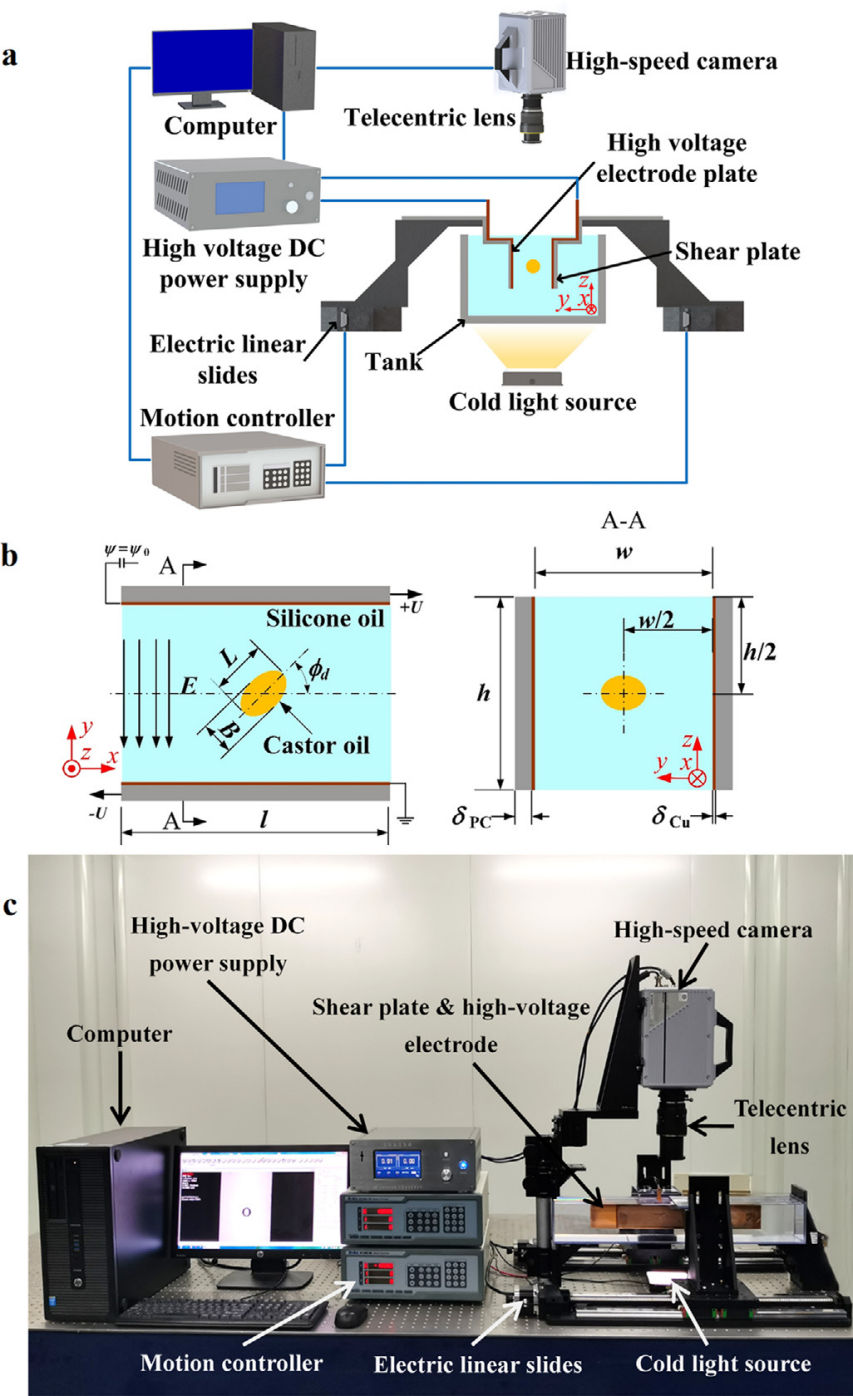
**Experimental setup**. (a) Schematic diagram of the experimental system. (b) Schematic of a droplet subjected to a uniform DC electric field and a shear flow field. (c) Photograph of the experimental apparatus.

The experimental system is composed of a high-speed visualization measurement system, an electrically-driven shear system, and a controllable high-voltage power supply system, as shown in Fig. 1a, c. The electrically-driven shear system contains two high-precision electric linear slides (Zolix TSA400-B), two controllers (Zolix SC300-3B), two parallel shear PC plates with plane sizes of 400 mm× 50 mm and thicknesses of 5 mm, and a continuous-phase tank. Two copper plate electrodes with the same plane size and thicknesses of 1 mm were tightly adhered to the inner surface of the shear plate. Parallelism between the two plate electrodes was achieved by a multi-DOF micrometric adjusting platform, which was checked before each tested condition by ensuring that the distance *w* was equal to 20*a* (where *a* is the droplet radius) with a micrometer (the parallelism error was approximately 50 μm over the maximum displacement distance of one shear plate of 250 mm). The continuous-phase fluid was filled into a transparent tank made of polycarbonate (PC), which had dimensions of 600 mm × 100 mm × 100 mm. With this system, a shear flow with a constant shear rate *v* = 2*U*/*w* could be produced between the two plate electrodes by moving them in opposite directions at a constant velocity ***U*** = (± 50 mm/s, 0, 0). The two copper plate electrodes were part of the high-voltage power supply system, which supplied an automatically-controlled DC high-voltage power (model: P303M1E, with a maximum output voltage of 30 kV and an accuracy of ± 10 V). These two electrode plates were connected to the voltage output terminal of the high-voltage power supply and ground, providing a uniform DC electric field between the plates. The transient deformation behaviors of the droplet were monitored by a high-speed visualization measurement system utilizing a high-speed camera (Photron FastCam SA2) coupled with a telecentric lens (Navitar1-50487) and were recorded in the computer. An LED light source (Leda F03-09) with cross-sectional dimensions of 100 mm × 100 mm, a power of 9 W and a light intensity of 900 lm was used to illuminate the observation region; the LED source had insignificant thermal effects on the flow field and droplet deformation in the current experiment.

### 2.2 Experimental procedure

In this work, the electrohydrodynamic deformation behaviors of a droplet under a pure DC electric field and a pure flow field were investigated, and the deformation characteristics of droplets subjected to these two fields were studied. Specifically, utilizing an accurate micropipette installed on a micromanipulator, adroplet with a radius of *a* = 0.5 mm was injected into the centerline of the ambient fluid (as shown in Fig. 1b). Then, the high-speed camera was activated to record the droplet deformation behaviors before a single field or a combined field were applied to the droplet. In particular, in the experiment on the electrohydrodynamic deformation of a droplet in a combined DC electric field and shear flow, a pure shear flow field was first applied until the droplet deformation reached a steady state, and then a DC electric field was superimposed until the droplet deformation reached another steady state.

Recognizing the internal flow of the droplet during deformation is also helpful for understanding droplet electrohydrodynamics in the presence of external physical fields [23,35]. Thus, digital particle image velocimetry (DPIV) [36,37] was used to quantify the internal fluid flow of the deformed droplet. In this work, polyamide spherical particles (PSPs, Btfluid 100063-1) with a density of 1.03 g/cm^3^ and a diameter of 10 μm were uniformly dispersed in the droplets. Based on repeated pretests, a 0.04 wt% PSP mass percentage concentration was appropriate and adopted in the current experiments, allowing the fluid flow in the deformed droplet to be traced well with negligible effects on the interface properties. In this way, the trajectory of the particles could be clearly observed and recorded by the high-speed visualization measurement system (the imaging frequency was 50 fps). Based on the frequency domain cross-correlation method [36], the internal fluid flow of the deformed droplet could be quantitatively derived from analyzing the particle position frame by frame.

### 2.3 Materials

Silicone oil (Shin-Etsu KF-96) and castor oil (Aladdin) were selected for the continuous phase (subscript c) or the droplet phase (subscript d). Table 1 shows the physical parameters of the two fluids. Based on previous results [20,22,38], the change in the physical properties of silicone oil and castor oil due to an external electric field can be ignored in the current study.

**Table 1:** **Physical parameters of the materials used in the current experiment**.

Medium	Density *ρ* (kg/m^3^)	Permittivity *ε* (F/m)	Conductivity *κ* (S/m)	Viscosity *μ* (Pa·s)	Interfacial tension*γ* (mN/m)
Castor oil	955	4.61 × 10^−11^	4.53 × 10^−11^	0.713	4.03
Silicone oil	971	2.39 × 10^−11^	4.32 × 10^−12^	0.969	

Furthermore, the Bond number $\mathrm{Bo}=\;\Delta \rho ga^{2}/\gamma \ll \;1$, where *γ* is the interfacial tension, *a* is the droplet radius, *g* is the gravitational acceleration, and Δρ is the density difference between the two fluids, indicates that in the current experiment, the effect of buoyancy caused by the density difference between the working fluids can be ignored, especially when compared with the action of the shear flow field and the DC electric field. In addition, the ambient temperature during the experiment was kept at 25 ± 0.5°C.

### 2.4 Deformation parameters definition

It should be noted that the Reynolds number of shear flow is far less than 1 × 10^−4^ (*Re* = *a*^2^*ρv*/*μ*_c_, where *μ*_c_ is the dynamic viscosity of the continuous phase), which indicates that the experimental conditions are well within the range of Stokes flow and that the inertial force can be ignored. Therefore, in pure shear flow, droplet deformation is mainly dominated by the competition between the viscous shear force and interface tension. Accordingly, the dimensionless hydrodynamic capillary number *Ca* was adopted to represent the ratio of the viscous force to the interfacial tension, which is described as *Ca = μ*_c_*va*/*γ*. Under the action of an unconfined pure shear flow or combined shear flow and DC electric fields, the suspended droplet deforms into an ellipsoidal shape. The deformation degree can be expressed by the deformation parameter [21]:

(1) $$D=\frac{L-B}{L+B}$$

where *D* is the deformation parameter and *L* and *B* are the major and minor axes of the ellipsoidal droplet, respectively (Fig. 1b). In addition, the tilt angle, *ϕ_d_*, is defined as the angle between the major axis of the deformed droplet and the positive *x*-direction (Fig. 1b). On the other hand, under the action of a pure DC electric field, the droplet deformation mainly depends on the competition between the electric force and the interface tension. Hence, the dimensionless electric capillary number *Ca*_E_ = *ε*_c_*E*^2^*a*/*γ* is used to characterize the ratio of the electric field force to the interfacial tension, where *E* is the electric field strength and *ε* is the permittivity.

**Fig. 2:**
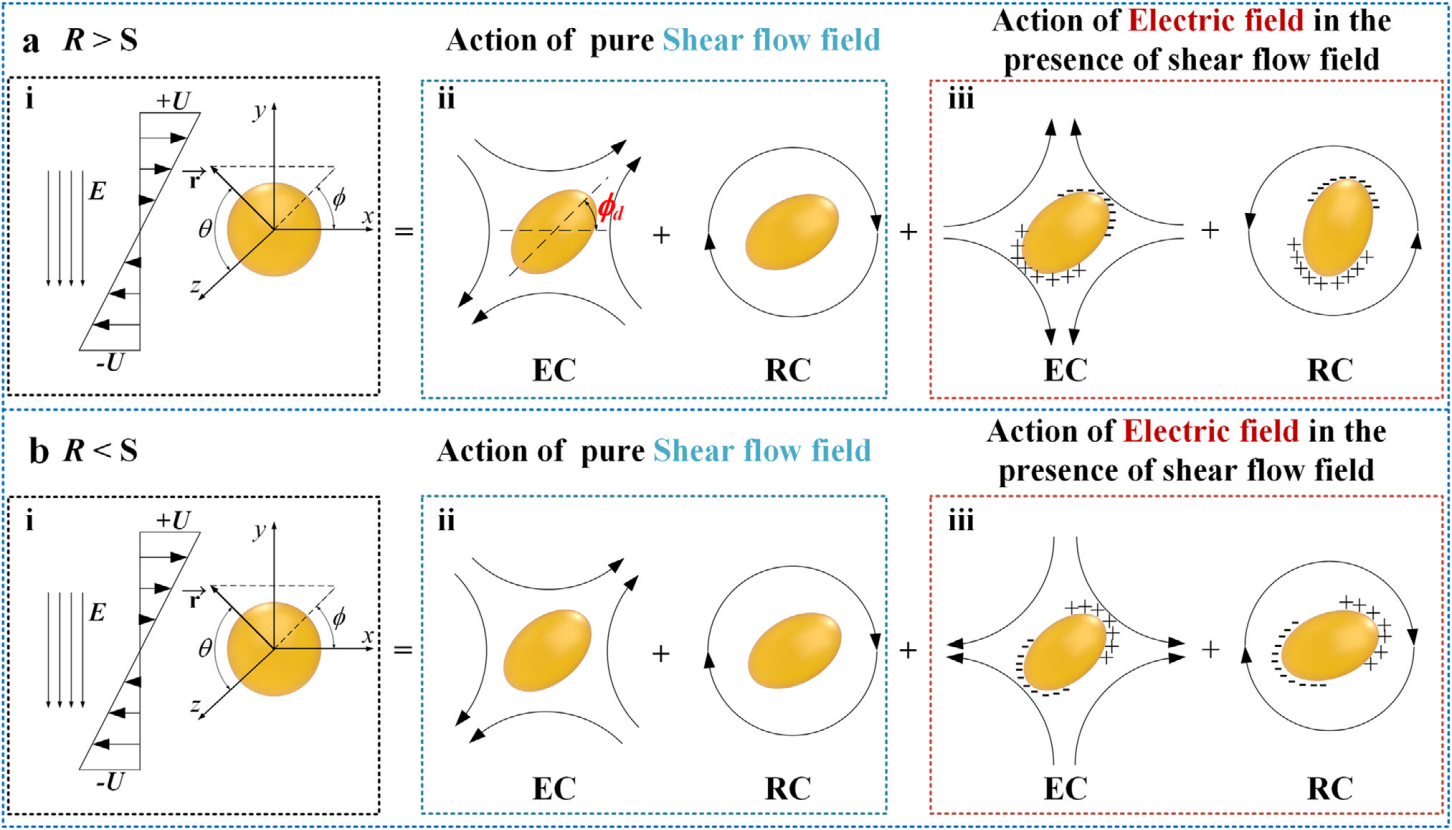
**The actions of shear flow and electric fields.**(a) When the droplet deforms into a prolate shape in a pure electric field under *R* > *S*. (b) When the droplet deforms into an oblate shape under *R* < *S*.

In addition, according to the leaky dielectric model, the surface charge distribution and the consequent deformation shape of a droplet in an electric field can be determined by the ratio of *R* to *S* (*R*/*S*) [20,23,25], where *R* denotes the conductivity ratio *R* = *κ*_d_/*κ*_c_ and *S* denotes the permittivity ratio *S* = *ε*_d_/*ε*_c_. In fact, *R/S* characterizes the ratio of the charge relaxation time of the continuous phase to that of the droplet. When *R* > *S* (i.e., when the induced charges inside the droplet move faster than those outside the droplet), the induced charges are attracted to the electrodes (Fig. 2a). As a result, the surface fluid moves from the equator to the poles (Fig. 2a), leading to prolate droplet deformation. In contrast, when *R* < *S*, the induced charges are attracted to the equator (Fig. 2b), generally deforming the droplet into an oblate shape. However, when *R* < *S,* for some special cases, the droplet deforms into a prolate shape [39]. Therefore, to comprehensively characterize the deformation shape of the droplet in a pure electric field, Taylor [23]introduced a deformation characteristic function:

(2) $$\Omega =R^{2}+1-2S+3\left(R-S\right)\left(3\lambda +2\right)/5\left(\lambda +1\right)$$

where *λ* is the viscosity ratio of the droplet phase to the continuous phase. Ω < 0 indicates oblate deformation, while Ω > 0 indicates prolate deformation. Because prolate droplet deformation under *Ω >* 0 and *R* < *S* is rarely achieved in real applications, only *R* > *S* (the castor oil droplet suspended in silicone oil) corresponding to prolate droplet deformation and *R* < *S* corresponding to oblate droplet deformation in a pure electric field were investigated in this study.

Moreover, in the current study, the electric Reynolds number *Re*_E_ = *ε*_c_*v*/*κ*_c_, which characterizes the ratio of the charge relaxation time to the convection time, is greater than 0.58, suggesting that the induced charges could not accumulate at the interface instantaneously and that the SCC cannot be ignored.

**Fig. 3:**
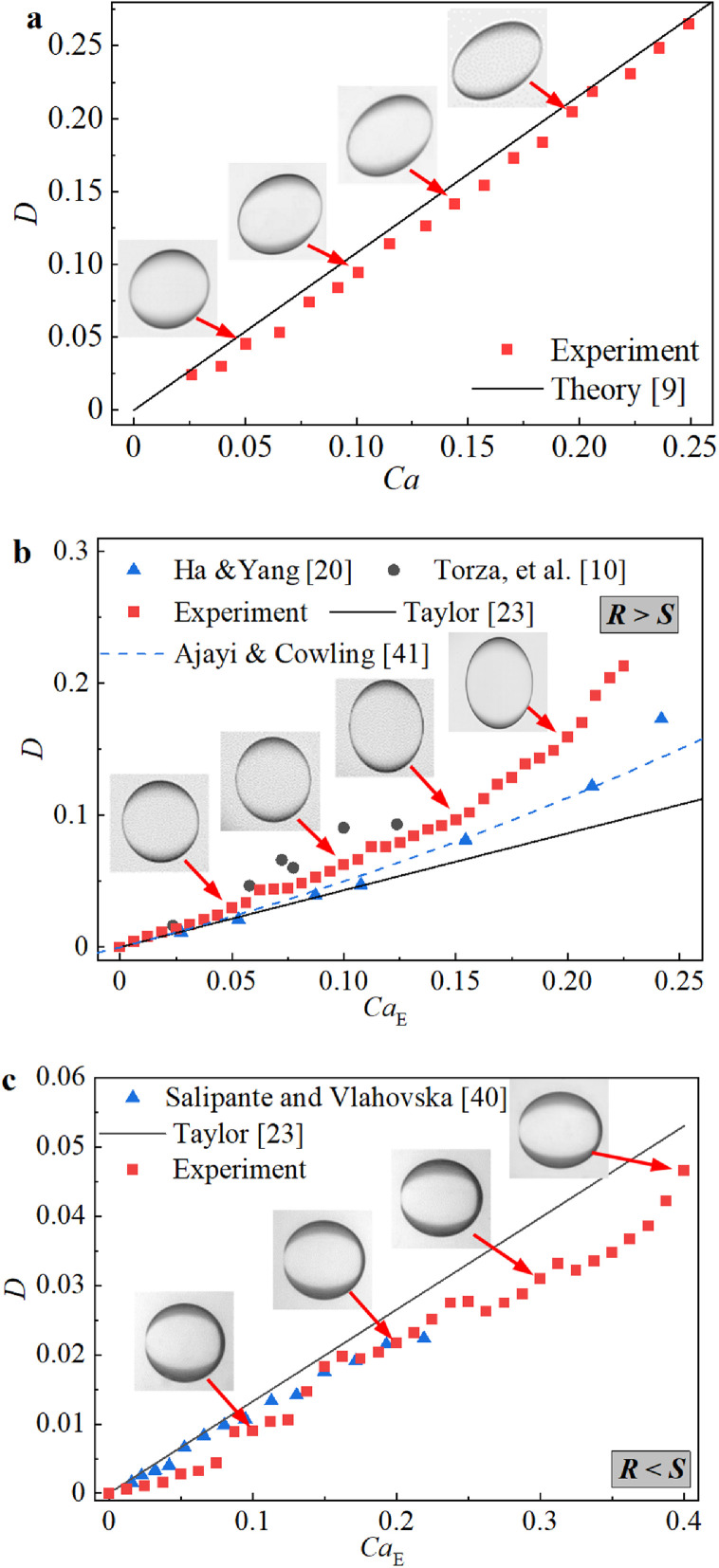
**Droplet deformation under pure shear flow field or pure electric field**. (a) Effect of *Ca* on *D* under the pure shear flow field. (b) Effect of *Ca_E_* on *D* under the pure electric field when *R* > *S*. (c) Effect of *Ca_E_* on *D* under the pure electric field when *R* < *S*.

In this work, in view of the few available effective experimental data on droplet deformation in a combined external field and electric flow, the deformation degrees of droplets in pure shear flow andpure DC electric fields were compared with those obtained by other experiments and classical theoretical predictions [9] to validate the reliability of the current experimental setup. As shown in Fig. 3, for the droplet deformation parameters under pure shear flow or a pure DC electric field, the experimental data obtained by the current setup are consistent with those obtained by other well-known experimental and theoretical methods [10,20,23,40,41]. Therefore, it can be concluded that the current results are both scientifically accurate and reliable.

## 3 Results and discussion

### 3.1 Droplet deformation morphology under *R* > *S*

**Fig. 4:**
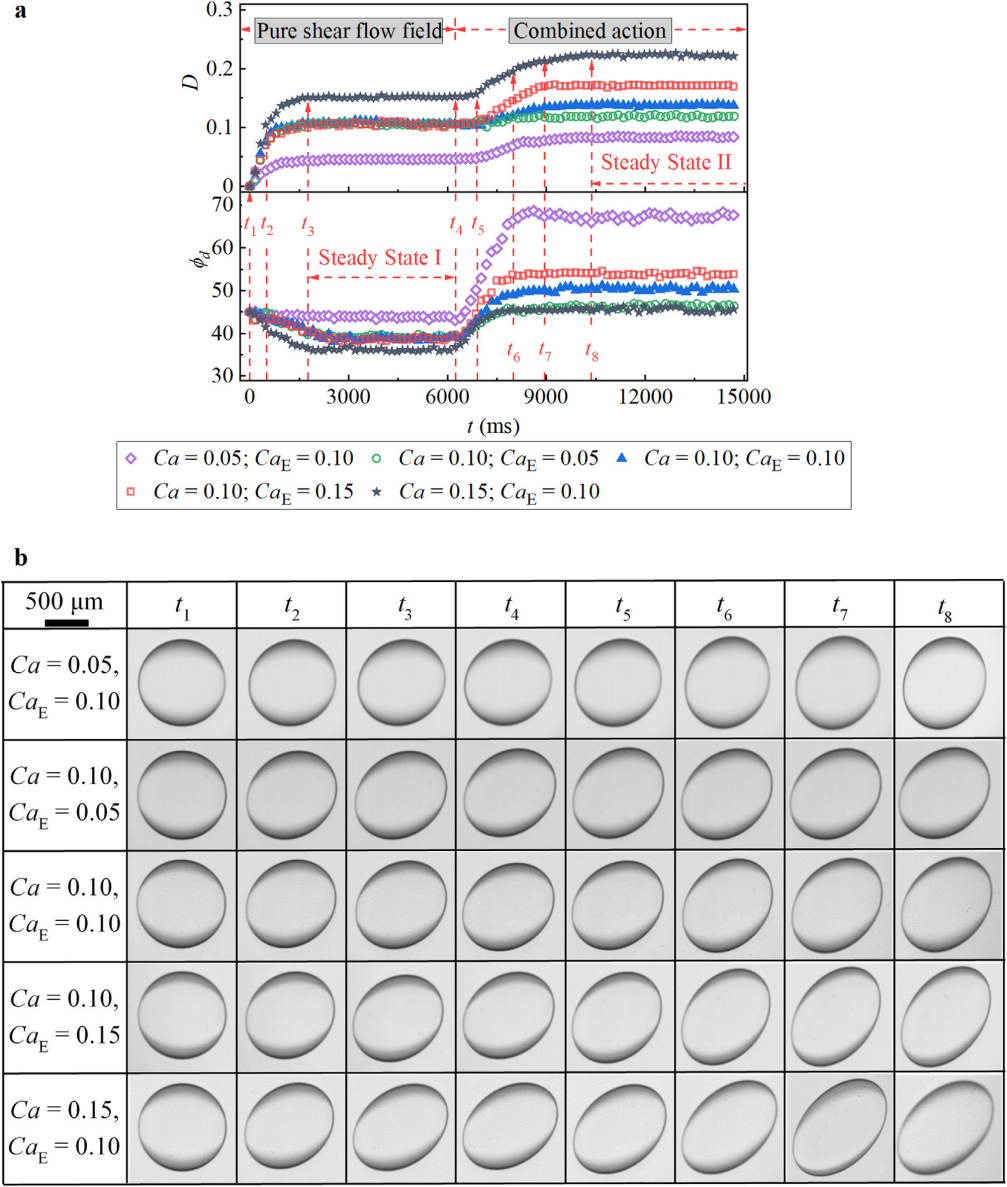
**Transient characteristics of droplet deformation under the combined action of the uniform DC electric field and the shear flow field when*****R*****>*****S***. (a) Evolution of the *D* and the *ϕ_d_* as a function of time. (b) Experimental phenomena of the droplet at the time corresponding to (a) and (b).

As mentioned in Section 2, to test the droplet deformation characteristics under a combined shear flow field and an electric field, a uniform DC electric field was imposed after steady deformation was attained in the pure shear flow field. Fig. 4 shows the whole droplet deformation process in the current experiment for different combinations of DC electric fields and shear flows. After a pure simple shear flow was applied to the spherical droplet, the spherical droplet became stretched toward the *x*-direction until it achieved a steady deformation state (Fig. 4). Correspondingly, *D* gradually increased with a gentle decrease in *ϕ_d_* until it reached a constant value (Fig. 4). Clearly, a greater *Ca* (i.e., a larger shear force) led to faster changes in *D* and *ϕ_d_*, which resulted in a larger *D* and a smaller *ϕ_d_*.

When the two fields were combined, the droplet deformation could be further enhanced under different *Ca*_E_ values, suggesting that when *R* > *S*, the electric force collaborates with the shear force (Fig. 4) and generally promotes droplet extension in the major-axis direction. In addition, the electric force always tends to rotate the deformed droplet in the direction of the velocity gradient, leading to a noticeable increase in the tilt angle *ϕ_d_* (Supplementary movie 1). In particular, under the action of shear flow at the same *Ca* value, increasing *Ca*_E_ step-by-step increased *D* (increasing *Ca*_E_ from 0.05 to 0.15 by Δ*Ca*_E_ = 0.05 at *Ca* = 0.1 in Fig. 4a), but without the action of shear flow, increasing *Ca*_E_ only induced linear changes in *D* within *Ca*_E_ = 0.15 (Fig. 3b). This indicates that the shear flow field amplifies the droplet deformation caused by the electric field. In fact, this phenomenon is mainly due to the additional SCC induced by the shear flow, which enhances the action of the electric field on the prolate deformation. On the other hand, increasing *Ca* by the same degree under a constant *Ca*_E_ also increases the absolute change in *D* (increasing *Ca* from 0.05 to 0.15 by Δ*Ca* = 0.05 at *Ca*_E_ = 0.10 in Fig. 4), implying that the additional SCC caused by the shear flow is enhanced by strengthening the shear flow on the droplet.

In addition, when the DC electric field was applied, the change in *ϕ_d_* (since *t*_4_ in Fig. 4a) appeared to occur earlier than the change *D* (since *t*_5_ in Fig. 4a), implying that the droplet first rotated and then deformed. This phenomenon could be explained by the characteristic competition of actions caused by the external shear flows and electric fields. As illustrated in Fig. 2a, the actions on the droplet, whether from the pure external shear flow or the electric field in the presence of a shear flow field, can be divided into an extensional component (EC) and a rotational component (RC), which stretch and rotate the droplet, respectively [25]. In particular, when *R* > *S*, the direction of the RC from the electric field is opposite that from the shear flow. As a result, when the electric field is combined with the shear flow, the tilt angle *ϕ_d_* changes immediately because the established equilibrium of the RC in the pure shear flow is broken. Notably, at this moment, the rotation of the droplet also weakens the original EC from the shear flow on the droplet due to the deviation from the optimum pose (Fig. 4). However, on the other hand, the new EC from the imposed electric field will facilitate droplet stretching. Due to these two competing effects, the droplet deformation degree (i.e., *D*) remains temporarily unchanged with initial droplet rotation.

**Fig. 5:**
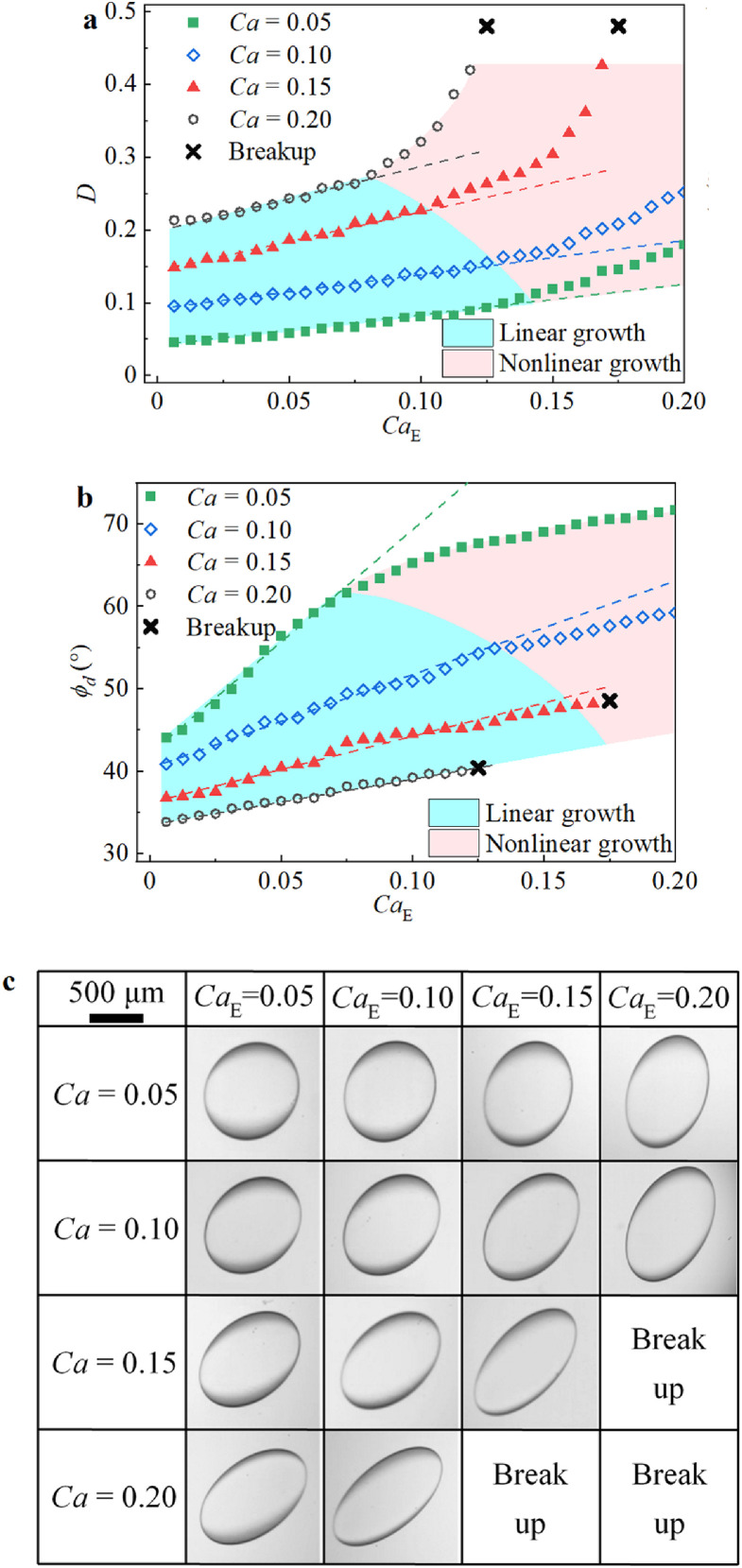
**Steady states droplet deformation characteristics under the combined action of a uniform DC electric field and shear flow field when*****R*****>*****S***. (a) *D*. (b) *ϕ_d_*. The straight lines are the fit lines of the corresponding data points. The blue background represents the linear region, and the pink background represents the nonlinear region. (c) Experimental phenomena of the droplet.

After the above transient process, due to the combined action of the two fields, the droplet will achieve steady deformation, with constant *D* and *ϕ_d_*. Fig. 5 summarizes the steady-state *D* and *ϕ_d_* values, as well as the real deformation profiles, under the combined action of the two fields. It can be clearly seen that for a given *Ca*, the enhancing actions of EC and RC with the increasing electric field (i.e., growing *Ca*_E_) result in increased deformation (i.e., increased *D*) and increased droplet rotation (i.e., increased *ϕ_d_*), respectively. In particular, the change in *D* with increasing *Ca*_E_ is characterized by an initial linear increase followed by a faster nonlinear increase, which is similar to the growth trend of *D* versus *Ca*_E_ in a pure electric field, as shown in Fig. 3b. The charges accumulated at the interface are clearly convected by the flow from the equator to the poles, which increases the surface charge density at the two poles. As a result, the normal component of the electric force increases, leading to increased nonlinear droplet deformation. In addition, as mentioned above, the SCC enhances prolate deformation while decreasing oblate deformation, which could explain the nonlinear change in *D*. In particular, the SCC could be further enhanced by the shear flow, especially when the shear flow is strong. Therefore, as shown in Fig. 5a, under a larger *Ca,* the nonlinear increase in *D* occurs at a smaller *Ca*_E_ and is also more pronounced. Similar to the variation trend of *D*, the change in *ϕ_d_* can be divided into linear-increasing and nonlinear-increasing regions (Fig. 5b). However, unlike the growth of *D* in the nonlinear region, the increasing rate of *ϕ_d_* in the nonlinear-increasing region is gradually reduced. The competition between the electric stresses and shear stresses on droplet rotation, which is quantitatively characterized by Eqs. 18-20, could be the reason for this phenomenon. Eqs. 18-20 show that, for the given fluid properties, the relationship between the droplet tilt angle and the ratio of *Ca*_E_ to *Ca* is an arctangent function. Consequently, under a given *Ca*, increasing *Ca*_E_ leads to a slower increasing rate of the tilt angle in the nonlinear-increasing region. In addition, a smaller *Ca* leads to a higher *Ca*_E_ to *Ca* ratio, and thus, the nonlinear-increasing region appears earlier, as shown in Fig. 5b. It should be noted that a similar phenomenon has also been reported by Ha and Yang [22].

### 3.2 Droplet deformation morphology under *R* < *S*

**Fig. 6:**
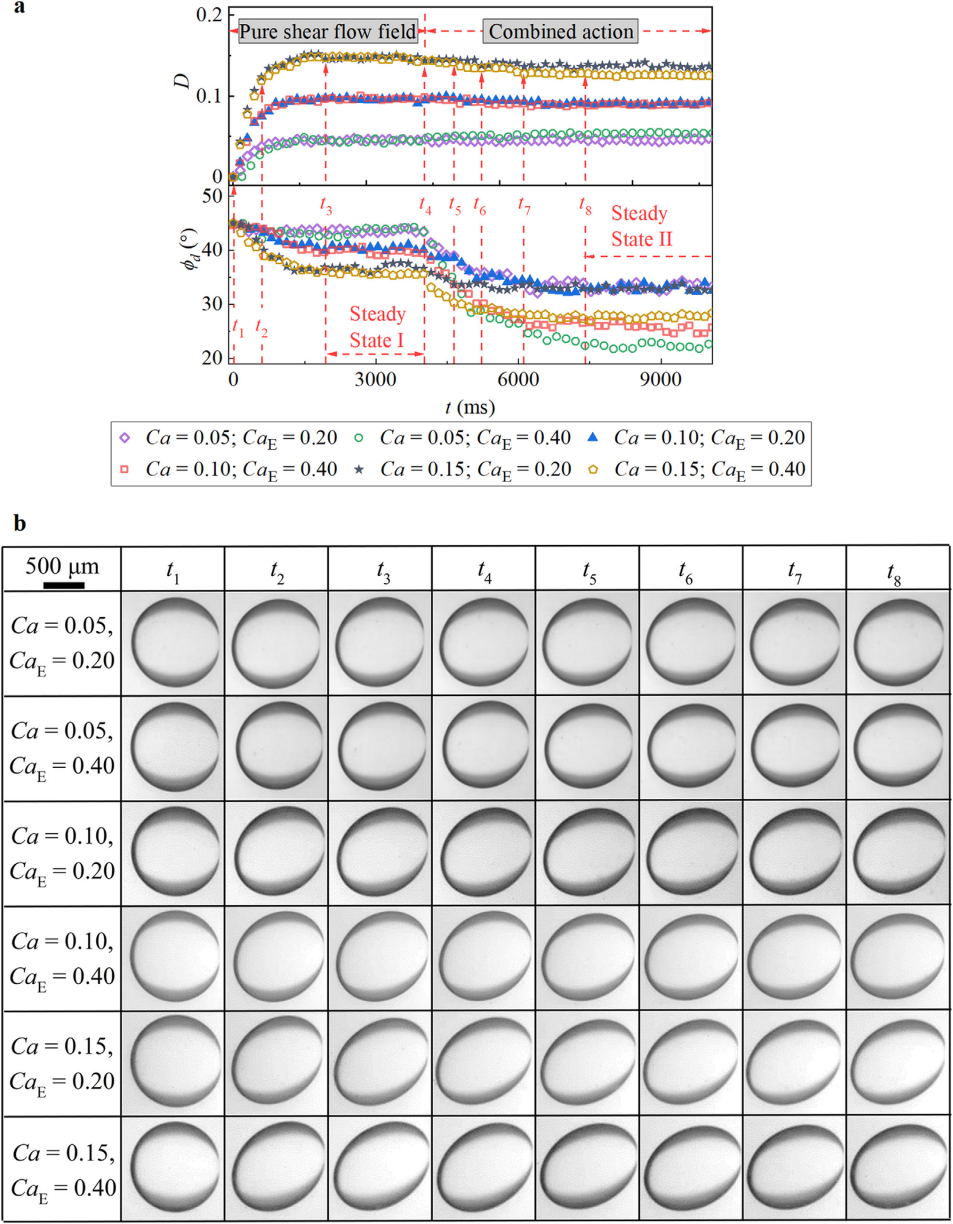
**Transient characteristics of droplet deformation under the combined action of a uniform DC electric field and shear flow field when*****R*****<*****S***. (a) Evolution of *D* and *ϕ_d_* as a function of time. (b) Experimental phenomena of the droplet at times corresponding to (a).

**Fig. 7:**
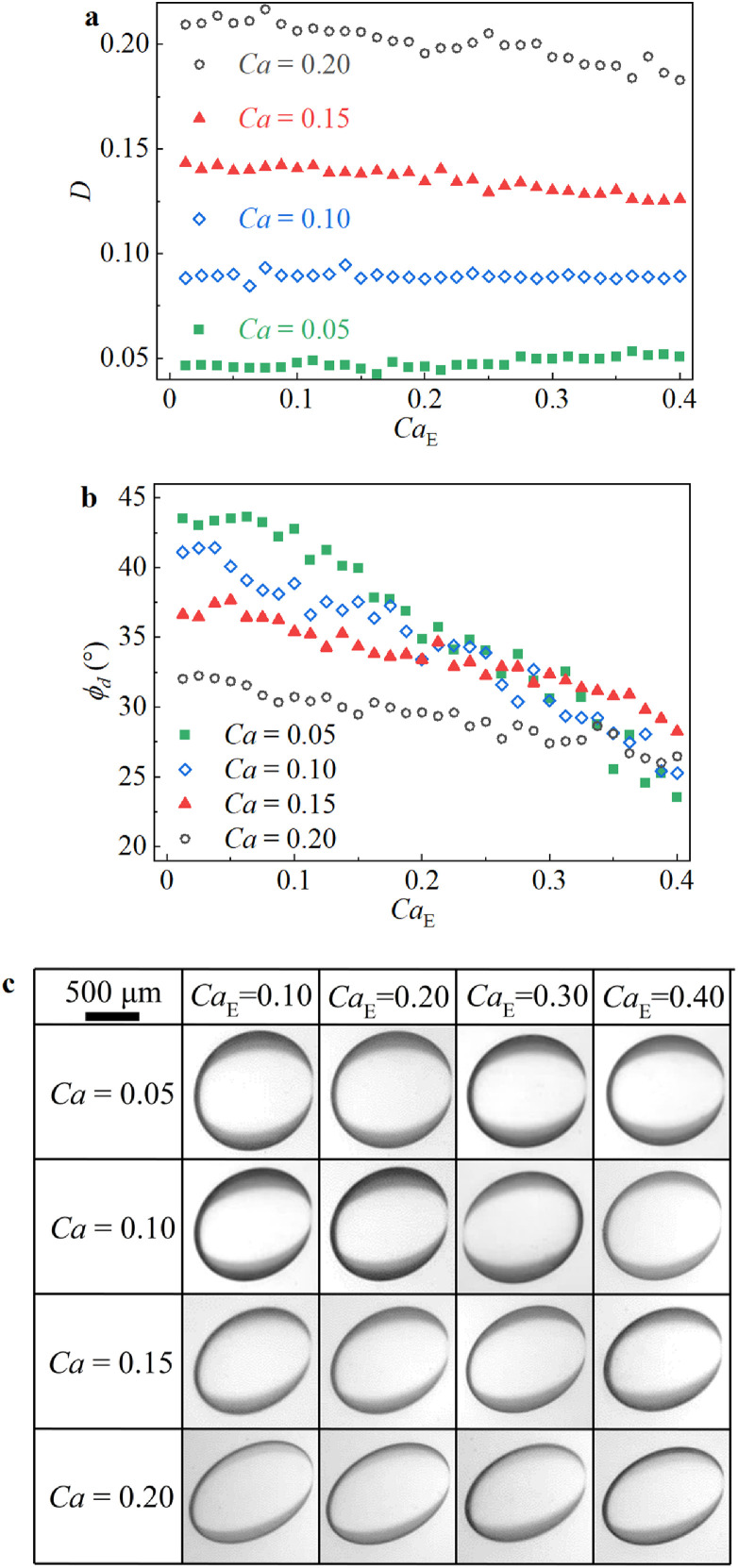
**Steady states droplet deformation characteristics under the combined action of a uniform DC electric field and shear flow field when*****R*****<*****S***. (a) *D*. (b) *ϕ_d_*. (c) Experimental phenomena of the droplet.

Compared with *R* > *S*, different deformation characteristics were observed when *R* < *S* (Fig. 3c). As shown in Fig. 6, when the electric field is combined with a weak shear flow under *R* < *S* (e.g., *Ca* = 0.05 in Fig. 6), droplet deformation could be enhanced slightly when *Ca*_E_ ranged from 0.2 to 0.4, suggesting that the electric force collaborates with the shear force and slightly promotes droplet extension in the major-axis direction (Supplementary movie 2). However, for *Ca*_E_ values ranging from 0.2 to 0.4, the droplet deformation parameter was almost unchanged when *Ca* = 0.1. Furthermore, when the external shear flow was enhanced (e.g., *Ca* = 0.15 in Fig. 6), the droplet deformation parameter was clearly reduced by increasing *Ca*_E_ from 0.2 to 0.4*.* These phenomena are also directly reflected in the steady-state droplet deformation morphologies depicted in Fig. 7.

In fact, this phenomenon is mainly due to the competition between the SCC and the electric stresses, where the former reduces oblate deformation and the latter stretches and rotates the droplet in the direction of shear flow. When *Ca* = 0.05, the latter dominates, leading to an increasing deformation trend as *Ca*_E_ increases. Furthermore, because the increasing *Ca* leads to a stronger SCC, which is almost balanced by the effects of electric stresses when *Ca* = 0.10, the deformation remains almost unchanged. On the other hand, the SCC becomes dominant by enhancing the shear flow (i.e., increasing *Ca* from 0.15 to 0.2), which reduces droplet deformation. In particular, *D* is lower at *Ca* = 0.2 than at *Ca* = 0.15, implying that the SCC is further strengthened by the shear flow (Fig. 7). It should be noted that because previous numerical simulation studies ignored the SCC [42], the combined action of the two fields is commonly assumed to increase droplet deformation monotonically, which is not the case.

On the other hand, when the electric field is combined with the pure shear flow at *R* < *S*, the tilt angle of the deformed droplet, *ϕ_d_*, decreases monotonically with increasing *Ca*_E_ under different *Ca* values, which is opposite to the phenomenon observed when *R* > *S*. This could also be explained by the characteristic cooperation of the actions of these two fields. As shown in Fig. 2, when *R* < *S*, the RCs from these two fields are in the same direction. As a result, when an electric field is applied, the tilt angle *ϕ_d_* decreases immediately (Fig. 6a). It should be noted that the general decreasing rate of *ϕ_d_* versus increasing *Ca*_E_ is reduced by increasing *Ca* (Fig. 6a), which could be attributed to the RC of the electric field contributing less to the inclination of the deformed droplet with increasing *Ca* than the shear flow field [38].

In addition, droplet deformation changes the shear stresses imposed on the droplet interface. Droplet elongation along the direction of the velocity gradient in shear flow effectively increases the torque from the shear flow, leading to increased viscous shear on the droplet [25,29]. On the other hand, droplet elongation along the direction of the shear flow results in a decrease in the viscous shear on the droplet. These results need to be investigated and demonstrated through further delicate experimental and numerical studies. The systematical critical conditions for droplet breakup also need to be studied further in the future.

### 3.3 Flow characteristics inside the deformed droplet

**Fig. 8:**
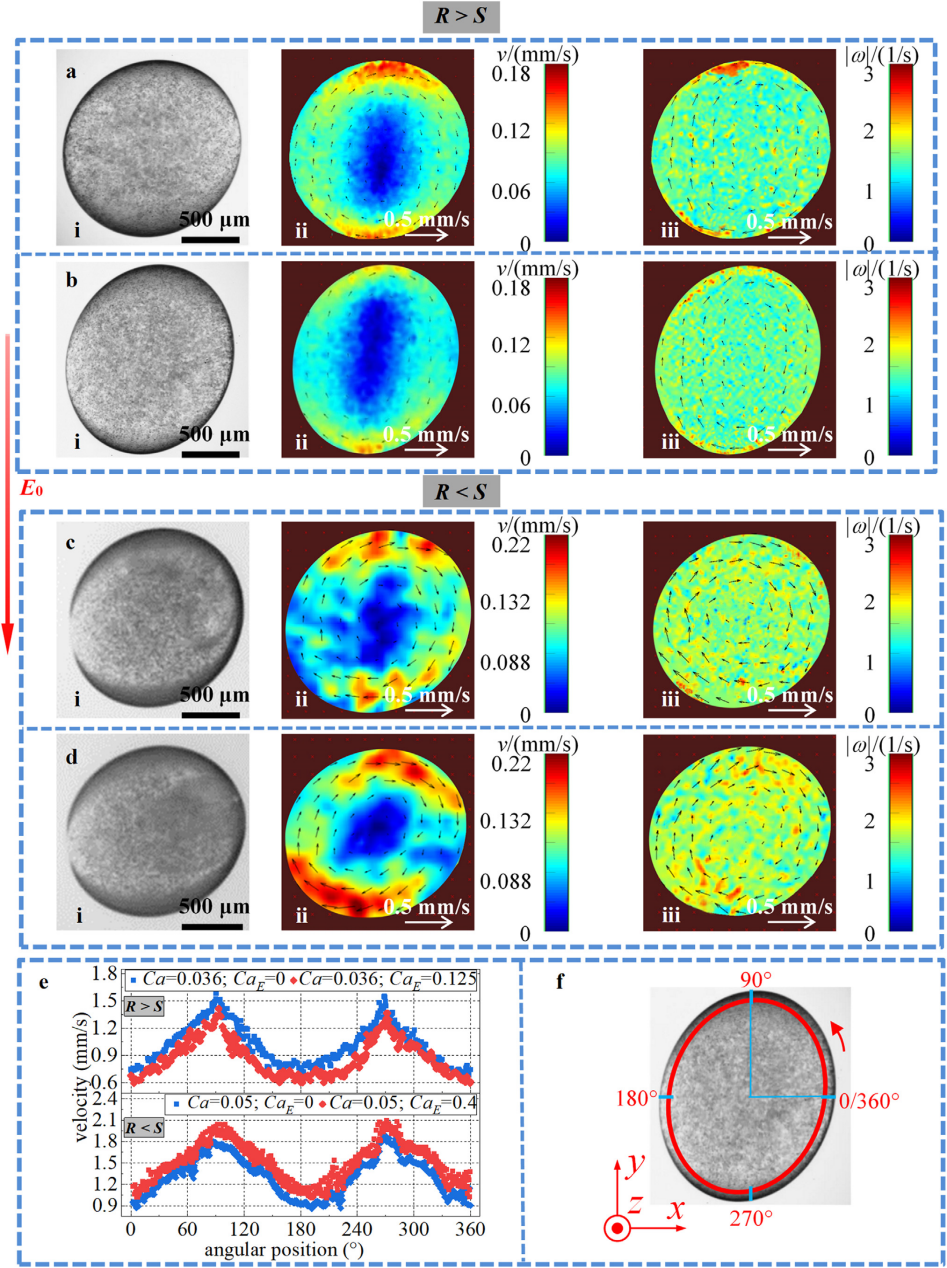
**The internal flow characteristics of the deformed droplet**. (i) Experimental images, (ii) PIV images of velocity magnitude contour maps, and (iii) vorticity magnitude contour maps of a droplet under the combined action of a shear flow field and a DC electric field. (a) *Ca* = 0.036, *Ca*_E_ = 0, *R* > *S*; (b) *Ca* = 0.036, *Ca*_E_ = 0.125, *R* > *S*; (c) *Ca* = 0.05, *Ca*_E_ = 0, *R* < *S*; (d) *Ca* = 0.05, *Ca*_E_ = 0.4, *R* < *S*. (e) Velocity distributions along a defined ellipse when *R* > *S* and *R* < *S*. (f) Schematic of the ellipse corresponding to (e).

In addition to the droplet morphology, the flow characteristics inside the droplet are important information for understanding of electrohydrodynamics underlying droplet deformation in a combined electric field and external shear flow field. Although numerical simulations can easily obtain detailed flow characteristics [43,44], there are still few quantitative experimental data on fluid flow information inside deformed droplets in a combined external shear flow and electric field. The typical flow characteristics inside the deformed droplet could be obtained quantitatively with the DPIV method in the current study, as shown in Fig. 8. It is clear that as a result of the competition among the shear force, electric force and interface tension, a vortex forms in the deformed droplet (Fig. 8), which could enhance the abovementioned SCC phenomenon. The vortex is nearly centrosymmetric, with the lowest velocity in the center, and its profile does not match the shape of the deformed droplet.

In particular, from the comparison between Fig. 8a, c (under pure shear flow) and Fig. 8b, d (under combined external shear flow and electric field), as well as the corresponding tangent velocity distributions along the droplet interface in Fig. 8e, it can be seen that for *R* > *S*, when a DC electric field is combined with an external shear flow, although droplet deformation increases, the absolute velocity of the closed vortical fluid in the droplet decreases, which can be attributed to the competition between the abovementioned RC from the external shear flow and electric field (Supplementary movie 3). As shown in Fig. 2a, when *R* > *S*, the RC due to the electric field is opposite to that due to the shear flow, which weakens the original action of the RC from pure shear flow and consequently decreases the internal vortical flow velocity [25]. On the other hand, for *R* < *S*, the internal cortical flow velocity increases because the RCs from the two fields are in the same direction (Fig. 2b and Supplementary movie 4). The vorticity magnitude contour maps inside the droplet are shown in Fig. 8, where |*ω*| represents the vorticity magnitude. It can be observed that the vortex inside the deformed droplet is not uniform, and the region near the shear plate has a higher |*ω*| than the center of the droplet. In addition, the electric field plays a minor role in vorticity in the presence of the shear flow, which further indicates that the internal vortex flow is dominated by the shear flow field.

### 3.4 The steady-state prediction models

Clearly, the deformation parameter and tilt angle are the most important parameters for characterizing the deformed droplet morphology in a combined electric field and shear flow field. Therefore, accurate predictions of these two parameters are regarded as a basis for guiding engineering applications [17], which is also an important concern of the current study.

#### 3.4.1 The steady-state prediction model of deformation parameter

The significance of quantitative prediction of the deformation parameter, *D*, has attracted great attention from previous researchers. As mentioned above, the droplet is a leaky dielectric [23], and the SCC [23] should be considered in the current study, which is consistent with the analytical solution by Mandal and Chakraborty [29] (called the MC model here) for predicting droplet deformation in a combined DC electric field and shear flow.

According to the MC model, both the Cartesian and spherical coordinate systems (*r, θ, ϕ*) are considered, with the origin at the droplet center, as shown in Fig. 2. The droplet interface *r_s_* can be expressed as:

(3) $$r_{s}\left(\theta ,\phi \right)=1+f\left(\theta ,\phi \right)$$

where *f*(*θ, ϕ*) is a function that represents the extent of the droplet shape deviation from sphericity.

For leaky dielectrics, charges are assumed to accumulate only at the interface [23]. Thus, based on the Laplace equation, the following equations govern the electric potentials *ψ* inside and outside the droplet:

(4) $$\left\{ \begin{array}{c} \nabla^{2}\psi_{d}=0\\ \nabla^{2}\psi_{c}=0 \end{array}\right.$$

Because the electric potential is continuous at the interface, the following equation is applicable:

(5) $$\psi_{d}=\psi_{c}$$

The balance of Ohmic conduction [25,39] and the SCC governs the surface charge distribution:

(6) $$\overset{\to }{n}\cdot (\nabla \psi_{d}R-\nabla \psi_{c})=-Re_{E}\nabla_{s}\cdot \left(q_{s}{\overset{\to }{u}}_{s}\right)$$

where $\overset{\to }{n}$ **=** ∇(*r* - *r_s_*)/∇(*r* - *r_s_*) is the outward unit normal, *q_s_* = $\overset{\to }{n}$ ⋅ (*S*∇*ψ*_d_ - *ψ*_c_) is the surface charge density at the interface, ∇_s_ = [∇ - $\overset{\to }{n}$($\overset{\to }{n}$ ⋅ ∇)] is the surface divergence operator [45], and ${\vec{u}}_{s}$ is the velocity at the droplet interface.

Under the condition of Stokes flow, the flow fields inside and outside the droplet can be characterized by the continuity equation and the Stokes equation:

(7) $$\left\{ \begin{array}{c} \nabla p_{d}\;=\;\lambda \nabla^{2}{\vec{u}}_{d},\;\;\;\nabla \;\cdot {\vec{u}}_{c}\;=\;0\\ \nabla p_{c}\;=\;\lambda \nabla^{2}{\vec{u}}_{c},\;\;\;\nabla \;\cdot {\vec{u}}_{c}\;=\;0 \end{array}\right.$$

The velocity field at the droplet interface is governed by no-slip and no-penetration conditions:

(8) $$\left\{ \begin{array}{c} {\vec{u}}_{d}\;=\;{\vec{u}}_{c}\\ {\vec{u}}_{d}\cdot \vec{n}={\vec{u}}_{c}\cdot \vec{n}\;=\;0 \end{array}\right.$$

In addition, at the droplet interface, the hydrodynamic tractions and the electric tractions can be balanced by the capillary force as [29]:

(9) $$\left(\tau_{c}^{S}+\frac{Ca_{E}}{\text{Ca}}\tau_{c}^{E}\right)\cdot \vec{n}-\left(\tau_{d}^{S}+\frac{Ca_{E}}{\text{Ca}}\tau_{d}^{E}\right)\cdot \vec{n}=\frac{1}{\text{Ca}}\left(\nabla \;\cdot \vec{n}\right)\vec{n}$$

where *τ*^E^ and *τ*^S^ are the electric and hydrodynamic stress tensors [29], respectively.

The mathematical model is two-way coupled and nonlinear. To obtain an asymptotic solution for the above mathematical model, it is convenient to use the perturbation method to express all the field variables [25,29]:

(10) $$\chi =\chi^{\left(0\right)}+Re_{E}\chi^{(Re_{E})}+\text{Ca}\chi^{\left(\text{Ca}\right)}+\cdots$$

where *χ*^(0)^ is the leading-order term [25], $\chi^{\left(Re_{E}\right)}$ represents the first contribution of the SCC, and *χ*^(^*^Ca^*^)^ represents the first contribution of shape deformation [29].

The associated Legendre polynomial $P_{n,m}$ can be used to express the electric potential [25], where cos *θ* is the argument, *n* and *m* are the degree and order of $P_{n,m}$ and $a_{n,m},{\hat{a}}_{n,m},b_{-n-1,m}$ and ${\hat{b}}_{-n-1,m}$ are unknown coefficients [29].

(11) $$\psi_{d}=\sum_{n=0}^{\infty }r^{n}\sum_{m=0}^{n}\left[a_{n,\;m}\text{cos}\left(m\phi \right)+{\hat{a}}_{n,m}\text{sin}\left(m\phi \right)\right]P_{n,m}$$

(12) $$\psi_{c}=\psi_{\infty }+\sum_{n=0}^{\infty }\frac{1}{r^{n+1}}\sum_{m=0}^{n}\left[b_{-n-1,m}\text{cos}\left(m\phi \right)+{\hat{b}}_{-n-1,m}\text{sin}\left(m\phi \right)\right]P_{n,m}$$

On the other hand, Lamb's general solution can be used to express the velocity and pressure fields inside and outside the droplet [46]:

(13) $${\vec{u}}_{d}=\sum_{n=1}^{\infty }\left[\nabla \;\times \left(\vec{r}\chi_{n}\right)+\nabla \Phi_{n}+\frac{n+3}{2\left(n+1\right)\left(2n+3\right)\lambda }r^{2}\nabla p_{n}-\frac{n}{(n+1)(2n+3)\lambda }\vec{r}\nabla p_{n}\right]$$

(14) $$p_{d}=\sum_{n=1}^{\infty }p_{n}$$

(15) $$1{\vec{u}}_{c}={\vec{V}}_{\infty }+\sum_{n=1}^{\infty }\left[\nabla \;\times \left(\vec{r}\chi_{-n-1}\right)+\nabla \Phi_{-n-1}-\frac{n-2}{2n\left(2n-1\right)}r^{2}\nabla p_{-n-1}+\frac{n+1}{n(2n-1)}\vec{r}\nabla p_{-n-1}\right]$$

(16) $$p_{c}=\sum_{n=1}^{\infty }p_{-n-1}$$

where *χ_n_, p_n_*, and Φ*_n_* are the growing spherical harmonics; *χ*_-_*_n_*_-__1_, *p*_-_*_n_*_-__1_, and Φ_-_*_n_*_-__1_ are the decaying spherical harmonics; and ${\overset{\to }{E}}_{\infty }$ and ${\overset{\to }{V}}_{\infty }$ represent the electric field and velocity conditions in the shear flow field far from the droplet, respectively [29].

The electric potential of the perturbation and the flow field are independent from each order when substituting the double asymptotic expansion in the above equations [29]. Due to the orthogonality of the associated Legendre polynomial *P_n_*_,_*_m_*, all the uncertain coefficients in the solutions can be determined. The drop shape can be characterized by the following asymptotic form [33, 47]:

(17) $$r_{s}=1+\mathrm{Ca}f^{\;\left(\mathrm{Ca}\right)}+\mathrm{CaR}e_{E}f^{\;(\mathrm{CaR}e_{E})}+Ca^{2}f^{\;(Ca^{2})}+\cdots$$

where *Caf* ^(^*^Ca^*^)^, *CaRe*_E_*f* ^(^*^Ca^*^)^, and $Ca^{2}f^{\left(Ca^{2}\right)}$ represent the first contribution of the shape deformation, the first contribution of the SCC, and the second-order contribution of the shape deformation, respectively [29]. According to Eq. 17, which is based on the MC model, the deformation parameter and tilt angle could both be obtained.

**Fig. 9:**
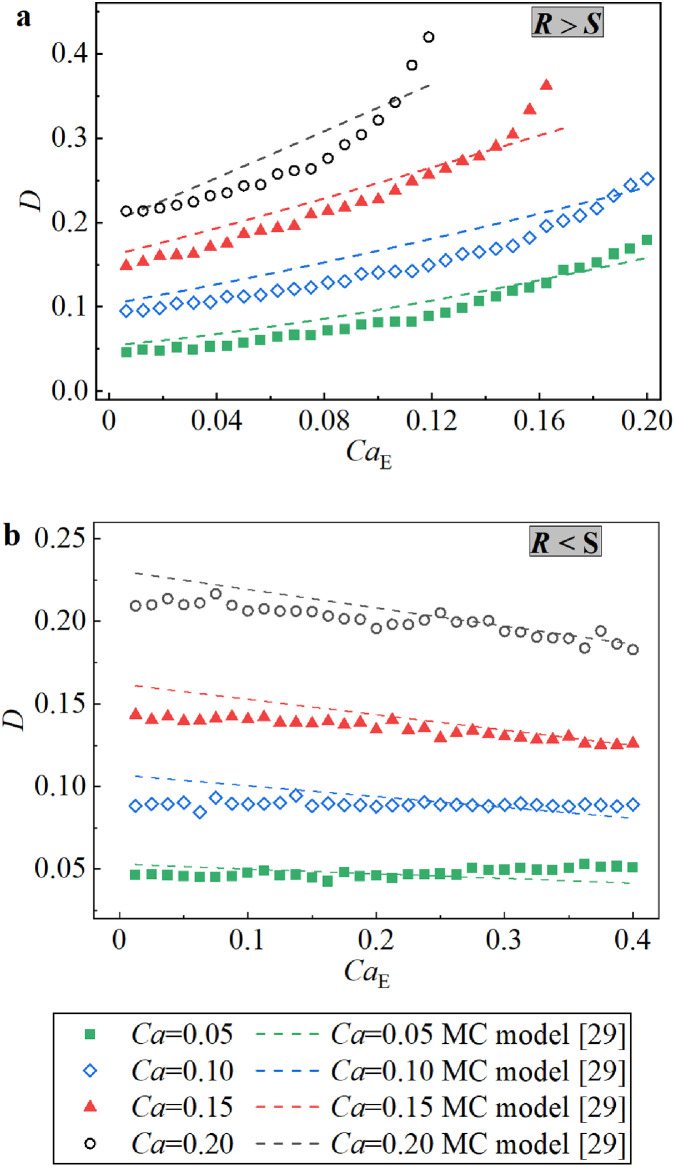
**Comparison between the MC model**[29]**and the experimental data on*****D*****under the combined action of a uniform DC electric field and shear flow field**. (a) When *R* > *S*; (b) When *R* < *S*.

However, the MC model has not yet been demonstrated via effective experimental results. Therefore, the predictions of the droplet deformation parameters by the MC model are compared with the present experimental data in Fig. 9. As shown, regardless of whether *R* > *S* or *R* < *S*, and despite the fact that many experimental cases are beyond the limits of the MC model (i.e., weak flow (Ca≪1) and weak SCC ($Re_{E}\;\ll \;1$)) [29], the predictions of the MC model agree well with the experimental data. In particular, when the external shear flow is weak (e.g., *Ca* = 0.05), the predicted and experimental results show remarkable agreement. Nevertheless, it must be pointed out that the predicted linear increase of *D* versus *Ca*_E_ under *R* > *S* in the MC model is inconsistent with the nonlinear increase of *D* versus larger *Ca*_E_ under strong shear flow (i.e., big *Ca*). In general, the MC model predicts *D* well in the range of the current work, with a mean absolute percentage error (MAPE) of 10.78% and a mean square error (MSE) of 0.014.

#### 3.4.2 The steady-state prediction model of the tilt angle

In 1962, when regarding the droplet as either a perfect conductor or an ideal dielectric, Allan and Mason [21] assumed that the electric stress imposed on the droplet, which is perpendicular to the viscous force provided by the external shear flow field, can be regulated by the Laplace equation. Accordingly, the curvature of a deformed droplet under a pure electric field can also be expressed by the Laplace equation. Therefore, utilizing trig transformation, the droplet deformation characteristics under the combined influence of an electric field and shear flow can be predicted by superposing the two individual Laplace equations of the electric force and viscous force, respectively. Then, the tilt angle of the deformed droplet can be expressed as:

(18) $$\phi_{d}\;=\;\frac{1}{2}\tan^{-1}(-\frac{D_{E}}{D_{S}})$$

where *D*_S_ and *D*_E_ are the deformation parameters under pure shear flow and the pure DC electric field, respectively.

Afterward, prediction models were further developed based on the Laplace equation, Stokes equation, and Ohmic conduction equation. To obtain the analytical solution of these equations, the perturbation method, Lamb's general solution and the associated Legendre polynomials are all utilized to express the field variables. Ha & Yang [22] first used this method to examine the influence of the membrane on the dynamic behaviors of a capsule subjected to the coupled action of an ambient linear flow and a uniform DC electric field. They stated that this method could also be applied to droplets. However, the exact equation for calculating the droplet tilt angle has not been published. A decade later, also using this method, Vlahovska [25] derived the expression of the droplet deformation parameter and the tilt angle in a combined shear flow field and an electric field, especially when the viscosity ratio of the droplet to the continuous phase is large. Compared to the prediction model proposed by Allan and Mason [21], this model has higher prediction accuracy and can account for the effect of the SCC. After that, Chakraborty's group [28,29] considered the effects of the SCC under weak flow conditions (*Ca* ≪ 1) and proposed a modified analytical solution for predicting droplet deformation behaviors under combined shear flow and electric fields. The differences among these models are due to the source terms of the governing equations; however, the fundamental principles are the same. Consequently, the prediction models for the tilt angle of the deformed droplet can be all described as:

(19) $$\phi_{d}=\frac{1}{2}\tan^{-1}\left(\frac{L_{S}}{L_{E}}\right)$$

where *L*_S_ and *L*_E_ can be expressed as [29]:

(20) $$L_{S}=-\frac{3}{16}\frac{Ca_{E}\Omega }{\mathrm{Ca}{\left(R+2\right)}^{2}}$$

(21) $$L_{E}=\frac{1}{48}\left(\frac{19\lambda +16}{\lambda +1}\right)$$

It is worth noting that when predicting the tilt angle of a deformed droplet, Eq. 18 and Eq. 19 have the same form. In particular, all these models are limited by weak flow and nearly spherical droplet shapes, with the assumption that the principle droplet deformation in the pure shear flow occurs at *ϕ_d_*_–S_ = π/4. Based on this assumption, the contribution of the electric force to the droplet orientation is coupled with *ϕ_d_*_-S_ when obtaining the tilt angle *ϕ_d_* under the combined action of an electric field and a shear flow field. However, this assumption deviates from reality when the shear flow becomes strong. Therefore, although these models agree well with the experimental data under weak flow conditions (see *Ca* = 0.05 as an example), they clearly deviate from the experimental data when the shear flow is strong (see *Ca* = 0.2 as an example).

**Fig. 10:**
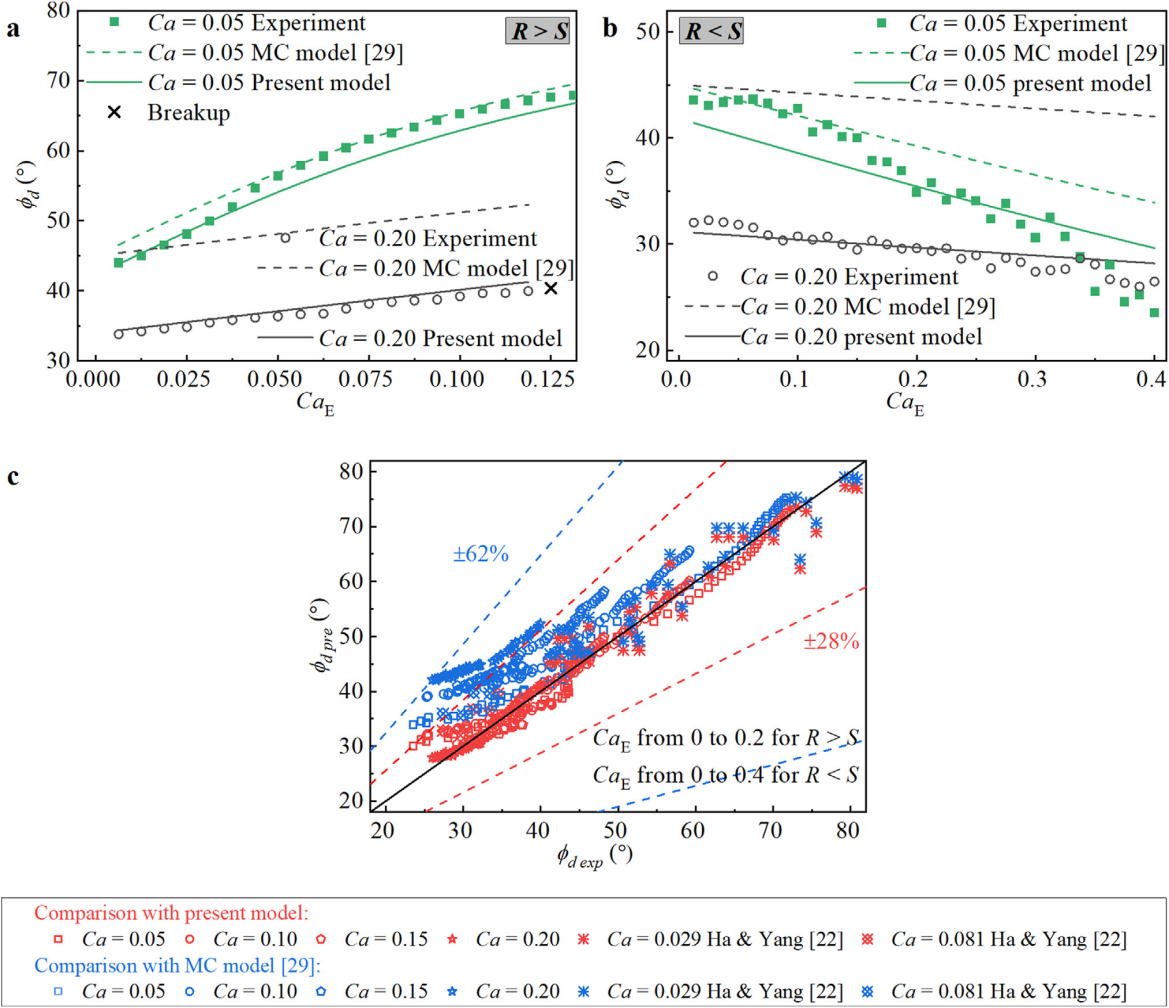
**Comparison between the MC model**[29]**, the present results, the experimental data and the results from Ha and Yang**[22]**on the steady-state tilt angle*****ϕ**_**d**_*. (a) When *R* > *S*. (b) When *R* < *S*. (c) Both *R* > *S* and *R* < *S.* The other parameters of the data when *Ca* = 0.029, as provided by Ha and Yang [22], are *R* = 10, *S* = 1.4, and *λ* = 0.7653. The other parameters of the data when *Ca* = 0.081, as provided by Ha and Yang [22], are *R* = 0.1, *S* = 0.71, and *λ* = 0.133.

Eq. 19 is based on the assumption that the deformation of the droplet is small when *Ca* ≪1. This is supported by the current experimental data, as shown in Fig. 10, where the predicted results from Eq. 19 shows good agreement with the experimental data under weak flow conditions (see *Ca* = 0.05 as an example). However, the predicted results clearly deviate from the experimental data when the shear flow is strong (see *Ca* = 0.2 as an example). This is mainly due to an undervaluation of the large inclination of the deformed droplet under strong shear flow (i.e., large *Ca*). It is worth noting that for droplet deformation in pure shear flow, a well-known formula based on Maxwell's model of elasticity [48] is accurate and feasible over a wide range of *Ca* values for predicting the tilt angle of the deformed droplet, which is expressed as [49,50]:

(22) $$\phi_{d}=\frac{\pi }{4}-\frac{\left(19\lambda +16\right)\left(2\lambda +3\right)}{80\left(1+\lambda \right)}\mathrm{Ca}$$

Accordingly, the different values between Eq. 22 and *ϕ_d__-_*_S_ is mathematically superimposed on Eq. 19 to reflect the large inclination of the deformed droplet under strong shear flow (i.e., large *Ca*), and Eq. 19 can be modified as:

(23) $$\phi_{d}=\frac{1}{2}\tan^{-1}\left(\frac{L_{S}}{L_{E}}\right)\;-\;\frac{\left(19\lambda +16\right)\left(2\lambda +3\right)}{80\left(1+\lambda \right)}\mathrm{Ca}\;\;\;\;\;\;\;\;\;\;\;\;\;\;\;\;\;\;\;\;\;\;\;\;\;\;\;\;\;\;\;\;\;\;\;\;\;\;$$

By using Eq. 23, the predicted *ϕ_d_* values agree well with the experimental data for both *Ca* = 0.05 and *Ca* = 0.20 in Fig. 10a, b. More importantly, as shown by additional comparisons between the prediction results and experimental data in Fig. 10c, Eq. 23 has a better prediction accuracy for the tilt angle of the deformed droplet in a combined electric field and external shear flow field. The prediction accuracy of *ϕ_d_* is remarkably enhanced, with an MAPE of 4.9% and MSE of 6.19, as opposed to the MC model, which has an MAPE of 22.2% and MSE of 75.48.

## 4 Conclusion

In summary, the detailed electrohydrodynamic deformation of a droplet, including the deformation degree (*D*) and tilt angle (*ϕ_d_*), in a combined DC electric field and shear flow field was studied via a visualization experiment. The internal flow characteristics of the deformed droplet were also quantitatively determined with the DPIV method. In addition, a prediction model for quantitatively representing *D* and *ϕ_d_* was investigated and modified based on the experimental data. The main conclusions are as follows:

• When a uniform DC electric field is combined with a shear flow field, the droplet tilt angle responds faster than the droplet deformation degree due to the competition between the extensional component (EC) and the rotational component (RC) of these two fields on the droplet.
• Combining the electric field with a pure shear flow field enhances droplet deformation when *R* > *S*, while it either enhances or reduces droplet deformation when *R* < *S*. Surface charge convection (SCC) plays a non-negligible role in enhancing and reducing droplet deformation for *R* > *S* and *R* < *S*, respectively.
• For *R* > *S*, the droplet is aligned more toward the shear flow direction under pure shear flow, while the electric field rotates the droplet in the direction of the electric field. For *R* < *S*, the two fields collaborate to rotate the droplet in the direction of the shear flow.
• Under the combined action of the two fields, an asymmetric vortex forms inside the deformed droplet. Because of the different interactions of the RCs from the two fields, the velocity of the internal vortex is reduced when *R* > *S* and enhanced when *R* < *S*.
• The available prediction models and the experimental data are used to predict *D*, and in particular, a modified model is proposed for improving the prediction accuracy of *ϕ_d_*, with a mean absolute percentage error of 4.9% and a mean square error of 6.19.

## Declaration of competing interest

The authors declare that they have no conflicts of interest in this work.

## Appendix Supplementary materials

Image, video 1

Image, video 1

Image, video 2

Image, video 3
